# Hemoadsorption as Adjunctive Therapy in Streptococcal Toxic Shock Syndrome

**DOI:** 10.7759/cureus.87667

**Published:** 2025-07-10

**Authors:** Gonçalo Miranda, Vasco Neves, Ricardo Tjeng, Pedro Lito

**Affiliations:** 1 Intensive Care Unit, Unidade Local de Saúde Cova da Beira, Covilhã, PRT; 2 Intensive Care Unit, Clinical Academic Center of Beiras (CACB), Covilhã, PRT; 3 Faculty of Health Sciences, University of Beira Interior, Covilhã, PRT

**Keywords:** adult intensive care unit, hemoadsorption, invasive group a streptococcus infections, septic shock (ss), streptococcal toxic shock syndrome (stss)

## Abstract

Group A *Streptococcus* (GAS) is a Gram-positive bacterium that can lead to invasive infections, such as necrotizing fasciitis or streptococcal toxic shock syndrome (STSS), a condition associated with high morbidity and mortality rates. We report the case of a 70-year-old male admitted to the intensive care unit with septic shock and multiorgan failure due to GAS bacteremia. The patient was treated with broad-spectrum antibiotics, intravenous immunoglobulin, and organ support measures, including vasopressors, renal replacement therapy, and hemoadsorption using the Jafron HA380 cartridge. This approach led to progressive clinical improvement and a full recovery. Hemoadsorption may have contributed to the resolution of shock by reducing circulating inflammatory mediators. This case supports its potential as an adjunctive treatment in STSS.

## Introduction

Group A *Streptococcus* (GAS) is a Gram-positive bacterium that typically causes mild illnesses such as tonsillitis and pharyngitis. In rare cases, it can lead to severe infections, including necrotizing fasciitis, bacteremia, septic arthritis, or pneumonia [1,2]. Thirteen percent to 15% of invasive infections are complicated by streptococcal toxic shock syndrome (STSS) [2].

Pathogenesis involves the release of streptococcal superantigens, leading to the massive release of cytokines and systemic inflammation, which can progress rapidly to life-threatening shock and organ dysfunction if not promptly recognized and treated [2,3].

Despite aggressive management with antibiotics, intravenous immunoglobulin (IVIG), and supportive care, STSS carries a high mortality, depending on organ involvement and treatment timing [2,3].

Hemoadsorption is an extracorporeal blood purification technique that removes circulating inflammatory mediators, such as cytokines, pathogen-associated molecular patterns, and damage-associated molecular patterns, using adsorptive cartridges or membranes integrated into a hemoperfusion or renal replacement therapy circuit [4]. Potential advantages of using hemoadsorption in the treatment of STSS include rapid reduction of circulating cytokines and streptococcal exotoxins, which may lead to improved hemodynamic stability, decreased vasopressor requirements, and mitigation of organ dysfunction. Case series have reported hemodynamic stabilization and improved metabolic parameters in septic shock patients, particularly when hemoadsorption is initiated early [5].

## Case presentation

A 70-year-old male with a history of hypertension and type 2 diabetes mellitus presented to the emergency department with a five-day history of productive cough, myalgias, asthenia, and fever. He was diagnosed with septic shock of presumed pulmonary origin and admitted to the intensive care unit (ICU). On admission, he exhibited multiorgan dysfunction, including circulatory failure, acute kidney injury (KDIGO stage 3), hyperbilirubinemia, and thrombocytopenia.

Initial management included empirical antibiotics (ceftriaxone and azithromycin), fluid resuscitation, vasopressor support (norepinephrine at 0.34 mcg/kg/min), and corticosteroids. Endotracheal intubation and mechanical ventilation were initiated within the first 24 hours due to worsening respiratory failure and shock.

On day 2, GAS was isolated from blood cultures, supporting the diagnosis of STSS. A worsening of the condition was observed, requiring an increase in vasopressor support (norepinephrine 1 mcg/kg/min and vasopressin 0.03 IU/h). Antibiotic coverage was escalated to include clindamycin, and IVIG was administered. Given persistent hemodynamic instability and renal failure, continuous venovenovenous hemodiafiltration was started alongside hemoadsorption therapy using the Jafron HA380 cartridge.

**Figure 1:**
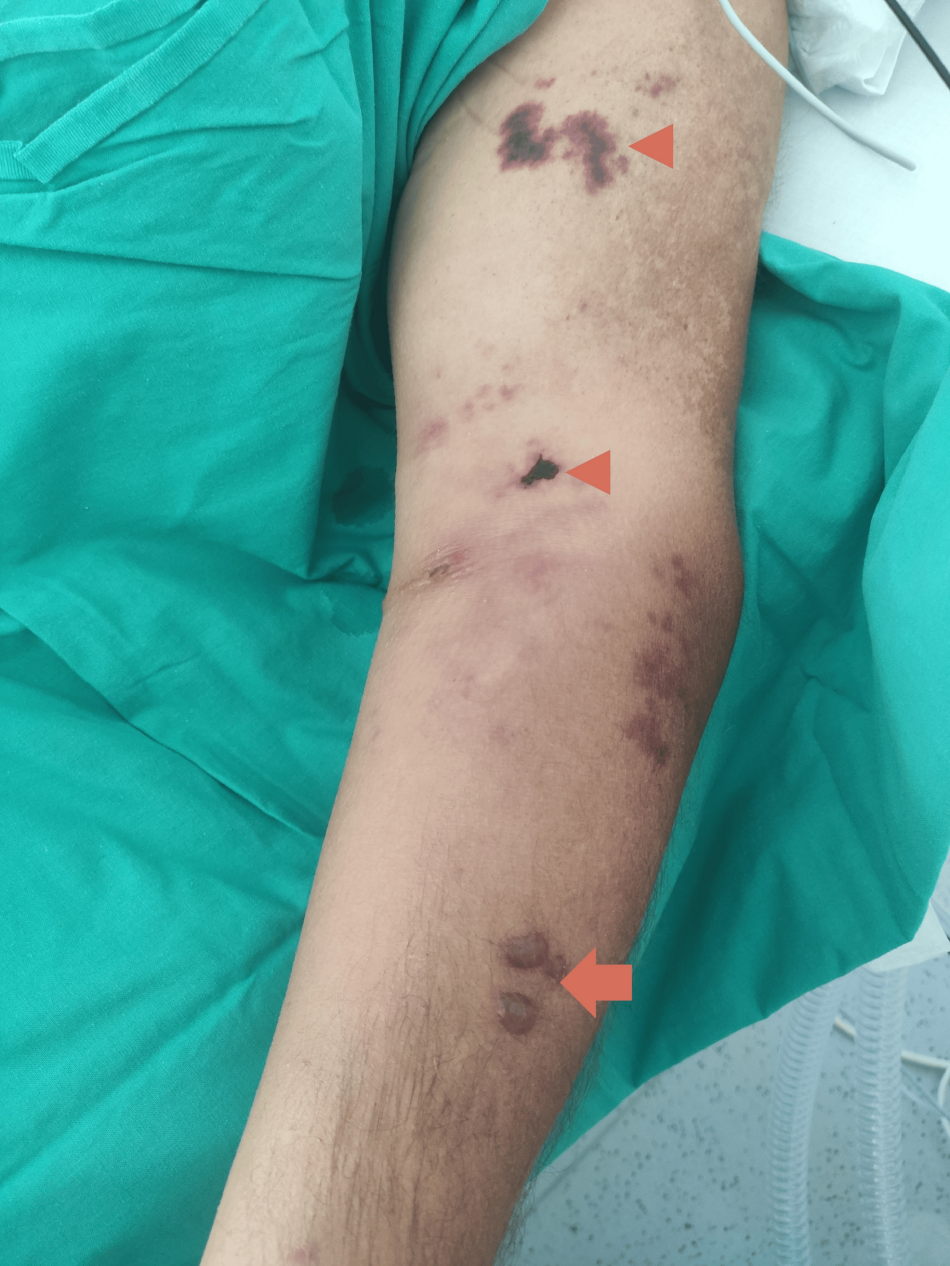
Left upper limb: edema, hemorrhagic bullae (arrow), and early skin necrosis (arrowheads)

**Figure 2:**
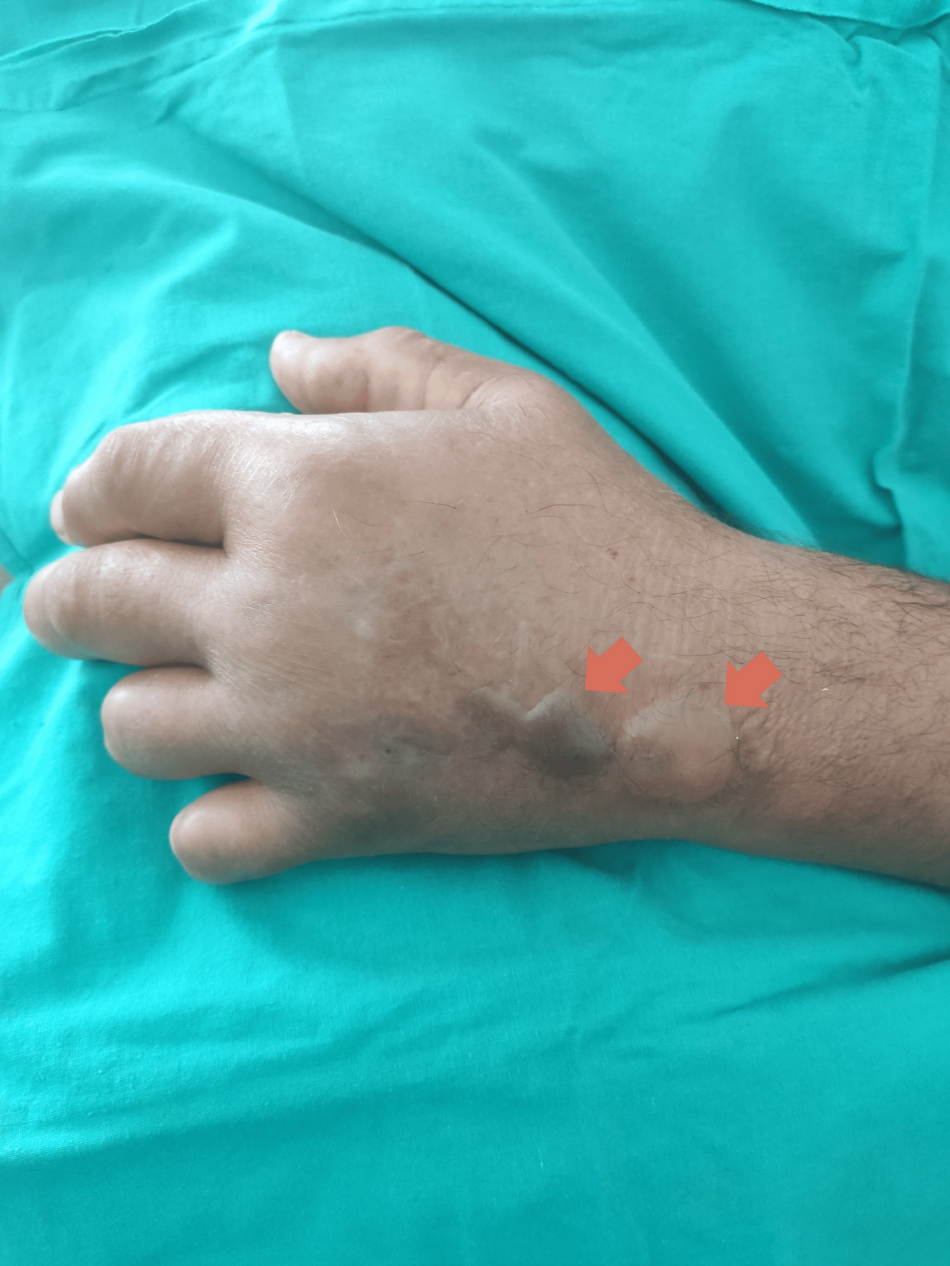
Left hand: extensive involvement with bullae (arrows)

**Figure 3:**
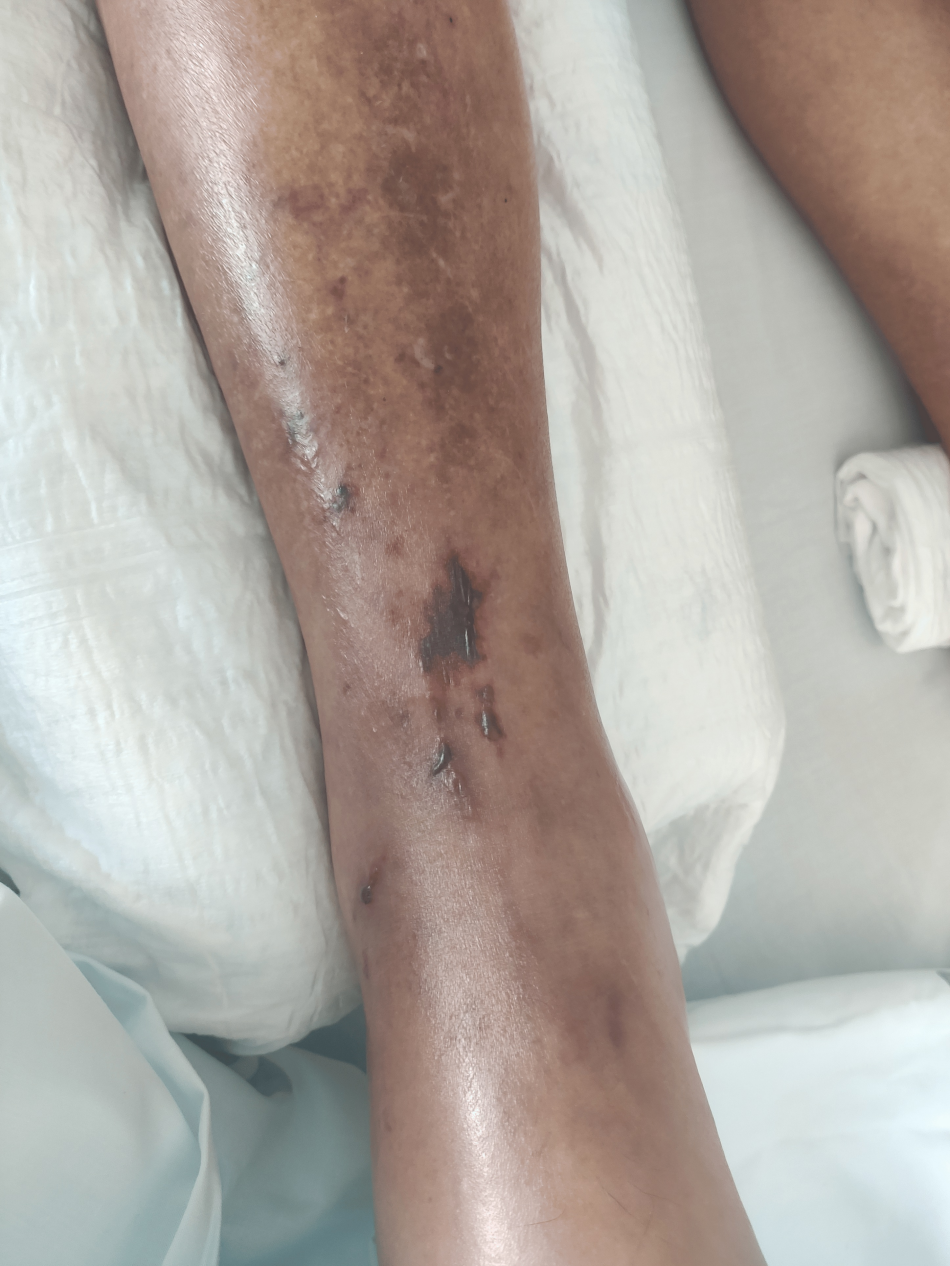
Right leg: soft tissue edema and hemorrhagic bullae

**Figure 4:**
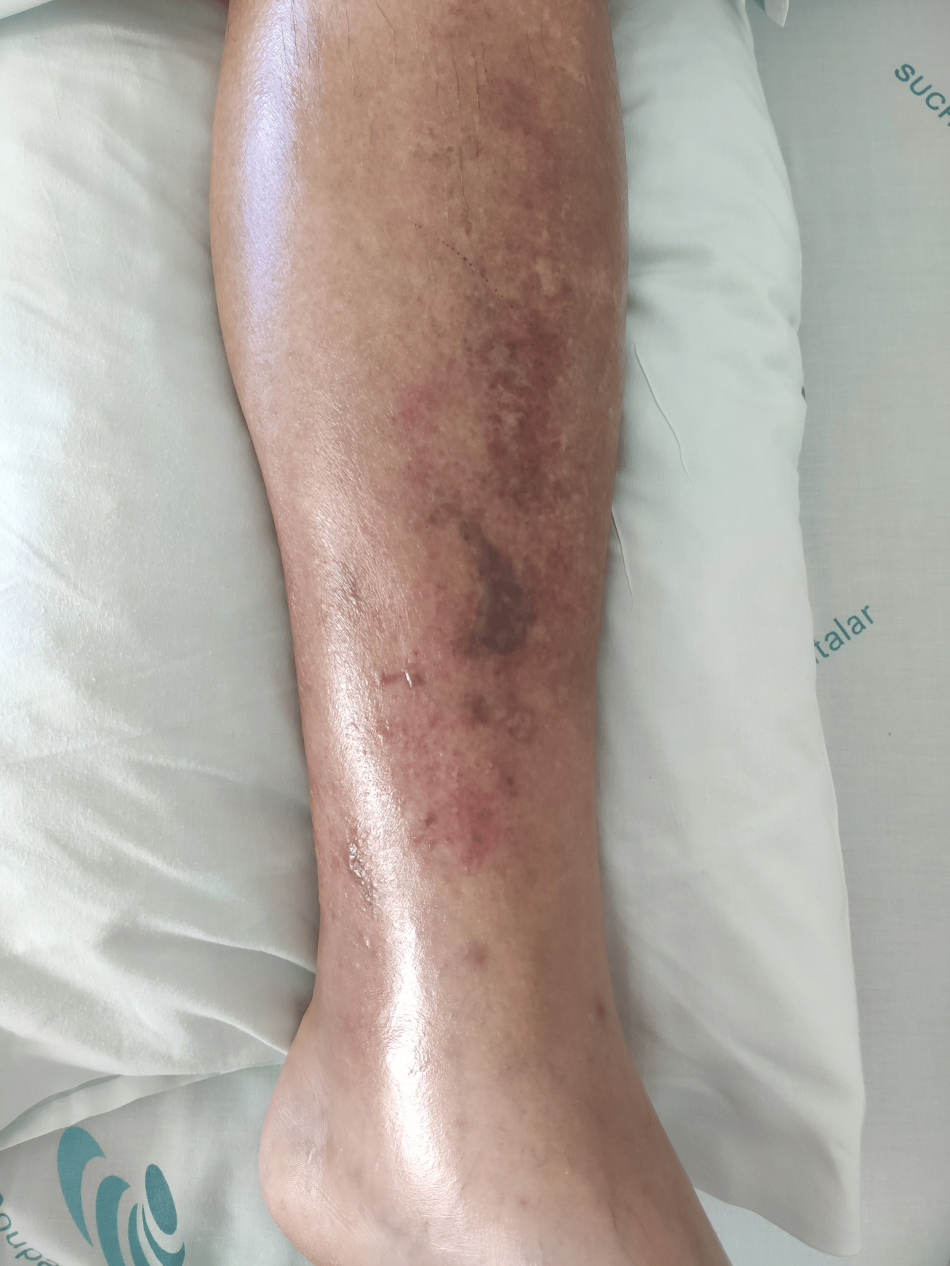
Left leg: areas of cutaneous necrosis and diffuse swelling

**Figure 5:**
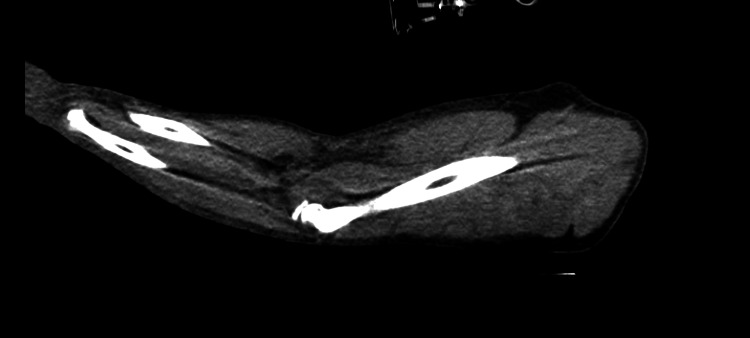
CT image of the left upper limb showing increased density of the subcutaneous tissues consistent with circumferential edema of the arm and forearm CT: computed tomography

In the first days of hospitalization, the patient developed progressive soft tissue involvement of the left upper limb (Figures 1-2) and lower limbs (Figures 3-4), with increasing edema, hemorrhagic bullae, and areas of cutaneous necrosis, raising concern for necrotizing fasciitis. A computed tomography (CT) scan of the left upper limb revealed densification of the soft tissues of the left arm and forearm, without evidence of fluid collections or gas. The axillary and brachial vessels were patent (Figure 5). These findings reinforced the severity of the invasive streptococcal infection and contributed to the decision to intensify therapeutic measures.

The patient’s condition improved markedly over the following days. Vasopressors were weaned off by day 4, renal replacement therapy was discontinued, and the patient was extubated on day 8. He was discharged from the ICU in stable condition and continued recovery on a medical ward.

## Discussion

This case illustrates the critical role of early recognition and aggressive supportive care in managing STSS. While the cornerstones of treatment remain timely antibiotic administration (including clindamycin to suppress toxin production) and IVIG to neutralize circulating superantigens, adjuvant therapies such as hemoadsorption are gaining interest [6-8].

Hemoadsorption aims to reduce the cytokine burden associated with STSS by physically removing inflammatory mediators from the bloodstream [4-8]. The Jafron HA380 cartridge is a resin-based device designed to adsorb a wide range of molecules, including interleukins, TNF-alpha, and other pro-inflammatory mediators. In this case, its use was temporally associated with hemodynamic improvement and renal recovery [9].

The presence and progression of cutaneous lesions, marked by edema, hemorrhagic bullae, and areas of necrosis, are indicative of severe invasive GAS infection and reflect a high systemic toxin burden. These skin manifestations are characteristic of STSS and have been associated with worse clinical outcomes due to their correlation with deeper tissue involvement and overwhelming inflammatory response [1-3]. In this case, the rapid deterioration of the skin findings further underscored the aggressiveness of the infection and the urgency of implementing multimodal therapy, including immunomodulatory strategies such as IVIG and hemoadsorption.

Although causality cannot be established in a single case, and spontaneous improvement with standard care cannot be excluded, the rapid stabilization observed after hemoadsorption initiation supports its potential benefit. This aligns with emerging literature on extracorporeal blood purification in sepsis and hyperinflammatory syndromes [4,5].

However, the 30th Acute Disease Quality Initiative Workgroup emphasizes that hemoadsorption remains an adjunctive therapy, with no demonstrated survival benefit in randomized controlled trials to date, and should not replace established first-line treatments such as antibiotics, IVIG, source control, and supportive care [10].

Furthermore, hemoadsorption appears safe in this context, with no significant adverse events reported in this patient. The potential benefits may be most pronounced in patients with high vasopressor requirements, as observed here, where traditional therapies are insufficient to control the inflammatory cascade [10].

## Conclusions

STSS is a life-threatening condition that requires prompt diagnosis and comprehensive management. Although antibiotic therapy and IVIG remain the cornerstones of treatment, hemoadsorption may represent a valuable adjunctive strategy in selected patients with refractory shock. This case contributes to the growing body of evidence supporting its potential benefits and underscores the need for further research in this area.
